# The role of Prefrontal Cortex in a Battle of the Sexes Dilemma involving a Conflict between Tribal and Romantic love

**DOI:** 10.1038/s41598-018-30611-6

**Published:** 2018-08-14

**Authors:** Isabel Catarina Duarte, Sónia Brito-Costa, Ricardo Cayolla, Miguel Castelo-Branco

**Affiliations:** 1 CIBIT, Institute of Nuclear Sciences Applied to Health, Faculty of Medicine, University of Coimbra, Coimbra, Portugal; 2 CNC.IBILI, Institute for Biomedical Imaging and Life Sciences, University of Coimbra, Coimbra, Portugal; 3 CDPH, Human Potential Development Center, IIA, Polytechnic Institute of Coimbra, Coimbra, Portugal; 4 DEGEIT, Department of Economics, Management, Industrial Engineering and Tourism, University of Aveiro, Aveiro, Portugal; 5 Porto Business School, University of Porto, Porto, Portugal

## Abstract

The neural basis of dilemmas involving decisions with profound affective impact, such as in romantic life, remains to be understood. The “Battle of the Sexes” is a paradigm from Game Theory that can be used to experimentally address such dilemmas. A form of in-group love, tribal love in football fans, provides the opportunity to study strong affective dilemmas when tribal and romantic love compete for hedonic decision-making. Here, we used for the first time a “Battle of the Sexes” dilemma using fMRI. We investigated, in 44 male football fans, the neural correlates of cooperative behaviour under conflicting choices in the context of romantic versus tribal love. We identified a critical functional segregation of prefrontal regions in affective decision-making. The orbitofrontal cortex signalled emotional appraisal of the dilemma. The medial anterolateral and the ventromedial prefrontal cortices reflected reciprocal cooperation instead of selfish engagement in football-related activities. The lateral portion of anterolateral prefrontal cortex was recruited during ultimate deliberation. In sum, emotional appraisal and rational choice reflected a contiguous functional parcellation in anterolateral prefrontal cortex: appraisal (medial) vs. choice (lateral region).

## Introduction

Moral dilemmas involving choice evaluations (appraisal) and abstract decision with no direct personal impact have been widely studied (for a review see^1^). However, the study of extreme personal dilemmas with strong conflicting affective options (as involving pair-bonding and love) and the role of distinct frontal regions in their resolution remains to be understood.

Love has been defined as a motivational and goal-directed affective mechanism to reinforce pair-bonding^2^, either in couples, families or tribes. Functional studies on romantic love have shown the recruitment of a core dopaminergic-related network, involving the ventral tegmental area, caudate nucleus, putamen, globus pallidus; and memory and emotion-related areas as the hippocampus and insula and anterior cingulate^3–7^. Other forms of inter-individual bonding, as ingroup love or tribal love, also showed to share similar neural underpinnings with romantic love^8^. This arousal and motivational state is important in the context of close relationships because it facilitates mutual cooperation, which has an evolutionary impact.

Long-term romantic partnerships involve repeated interactions, which reinforces the notion that cooperation based on pair-bonding and strong affective links is relevant for adaptive behaviour. For example, interpersonal altruistic behaviour plays an important role in reproductive success^9^. Cooperative behaviour implies inhibiting egocentric motives, while monitoring the affective state and partner’s intentions at multiple time scales. Mechanisms that weight long-term reciprocal benefits over the immediate individual gain, and the capability to inhibit conflicting prepotent actions that might disrupt the strength of affective links, are quite important in this context. Weighting affective and hedonic costs and benefits determines the ultimate affective and hedonic value of reciprocal interactions. The need to understand investment in long-term interactions is recognized in iterated game theory approaches, but even these are often based on single experiential interactions, and not the long-term ones related to romantic partnerships^10^.

Cooperation and rational decision making have often been studied using conflict and cooperation games as the prisoner’s dilemma or the stag hunt game^10–14^. Studies in the context of social interactions frequently involve monetary reward^10^, especially in the ultimatum game^15,16^. In some studies, the ‘social’ partner of the first player is a computer and in others, participants are meant to believe that they will play with humans, although they end up playing with computers as well^14^. This is known to influence the player’s actions in the ultimatum game as compared with the same game played with another human^16^. In imaging studies, it is also typical that the first contact with the social partner, if human, occurs only inside the scanner or just before the scanner session^17^. As the game progresses, the participant learns the partner’s intentions and strategies^14^. Some studies have reported a decrease in cooperation as the end of the game approaches^10,18^. One may intuitively expect that, since the term of that short ‘relationship’ is coming and the risks of defection became minor. Ecological designs are necessary to study conditional cooperation (i.e., the interaction between social partners based on reciprocal cooperative or non-cooperative behaviours) and to understand the role of values, such as fairness, in decision-making^19,20^. Commonly used neuroeconomic decision making paradigms have proven important to study the neural underpinnings of positive/negative or expected/unexpected outcomes^13^. However, these models, despite being essential to study the neural correlates of cooperative behaviour, do not account for the understanding of evolutionary ancient features of human interaction. These experimental designs can hardly elicit the neural processes of weighting the real consequences in a dyadic long-lasting relationship and the processes of acting sensibly as function of that affective investment.

The neural correlates of decision-making involving social tactics and strategic interactions are widely debated^10,11,21–25^. The role of dorsolateral prefrontal cortex (dlPFC) in such processes is well recognized^11,21–23^. The neural mechanisms underlying cognitive control during conflict processing are thought to involve also the anterior cingulate cortex (ACC)^10,11,24^. Moreover, strong reciprocal connections exist between dorsal ACC and dlPFC. The ACC is thought to detect the presence of conflict, which is signalled to the latter for ultimate decision^25^. Social decision-making involves the recruitment of networks associated with cognitive control, social cognition and reward processing^26–28^. Cognitive control and social cognition networks compute extrinsic incentives and trust signals, respectively, and play a top-down modulatory role over the reward processing system. Accordingly to this model, the decision on to cooperate (or not) is generated by the reward system as result of such top-down mechanisms. This model suggested by Declerck *et al*.^20^ proposes the integrative interaction of dorsolateral PFC, dorsal ACC and lateral orbitofrontal cortex (OFC) in the computation of the weights of extrinsic incentives.

**Figure 1:**
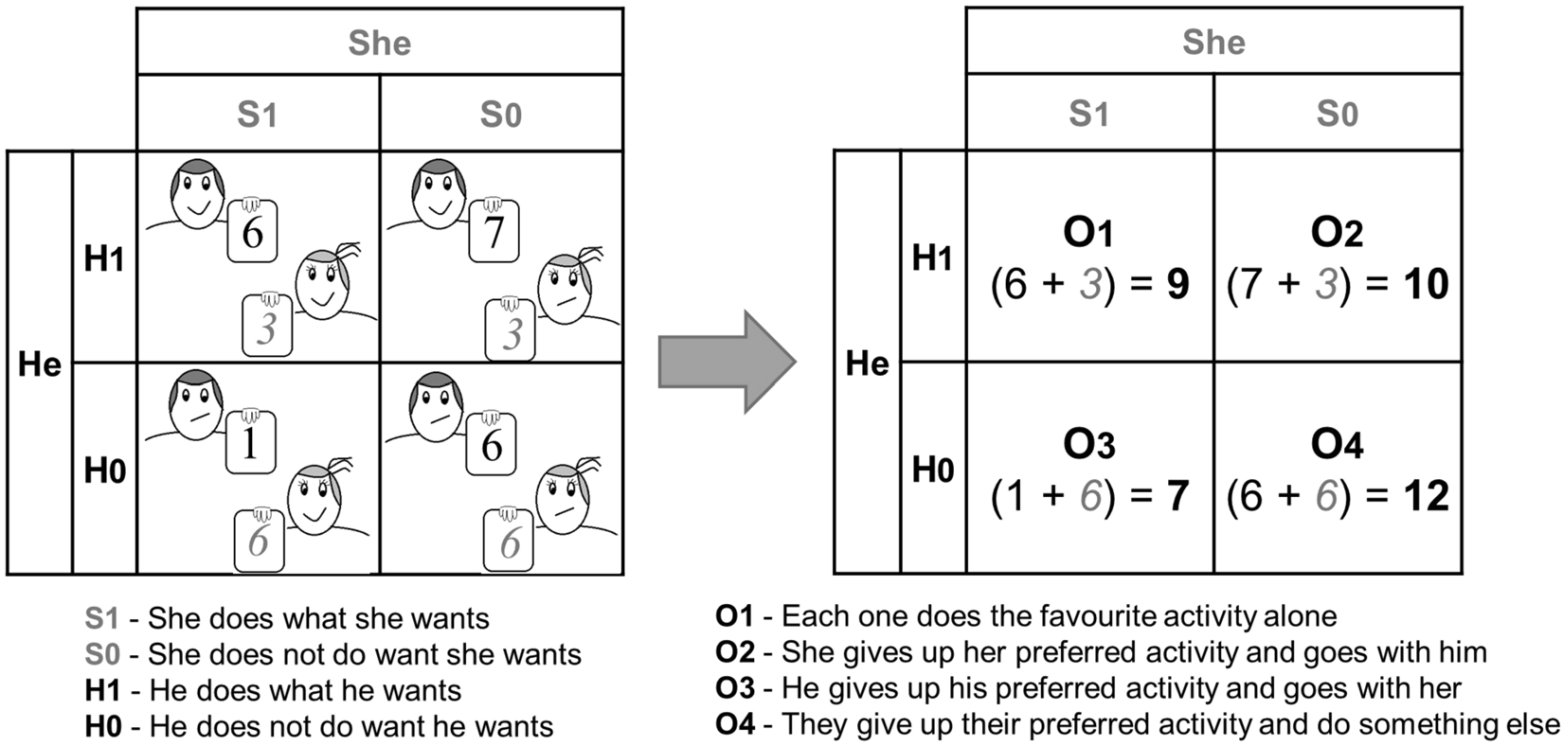
Illustration of a payoff matrix of a Battle of the sexes dilemma. The presented values are examples of what could be the answers on the 7-point Likert questionnaires concerning choice of activities. Note that one may prefer (and thereby score higher) an activity with the partner rather than doing it alone. Similarly, one can score to engage on the activity suggested by the partner higher than to go for his/her own option alone. Each quadrant in the payoff matrix corresponds to an option in the decision phase of the dilemma (O1-O4). The participant faced the four choice options, but was not directly exposed to the matrix values (which were extracted from personalized questionnaires and were used for further analysis). Here, option 4 (O4) would be the option corresponding to the highest joint payoff for the couple.

Here, we investigate the neural architectures involved in affective reasoning and decision-making in a battle of the sexes dilemma. We tested the relative role of limbic, reward and cognitive control networks in the presence of affective conflict, concerning the dilemma between egocentric and reciprocal couple related passionate motives. The chosen battle of the sexes paradigm was not previously addressed in neuroimaging research. We presented realistic dilemmas that were generated based on tailored questionnaires applied before scanning. Accordingly, participant personalized pay-off matrices could be created based on the personal history and emotional context (Fig. 1). To ensure that a strong dilemma was indeed present, we recruited a cohort of 44 neuropsychologically scored football fans^8^, engaged in a long-lasting relationship.

Experimental design (detailed in Supplementary Fig. S1) includes appraisal and deliberation periods. Dilemmas implied conflicting choices between football related activities (always involving an important match of the participant’s favourite team) and alternative options relevant to the participant’s partner.

## Results

### Behavioural results

For all dilemmas of each participant, we computed the payoff matrixes and verified the subsequent responses during neuroimaging. We found that 39.7 ± 30.1% (grand mean ± SD) of all choices gave preference to the highest outcome from the couple’s reciprocal perspective, while 60.3 ± 30.1% were options of lower outcome value for the couple as a dyad.

### Imaging results

**Figure 2:**
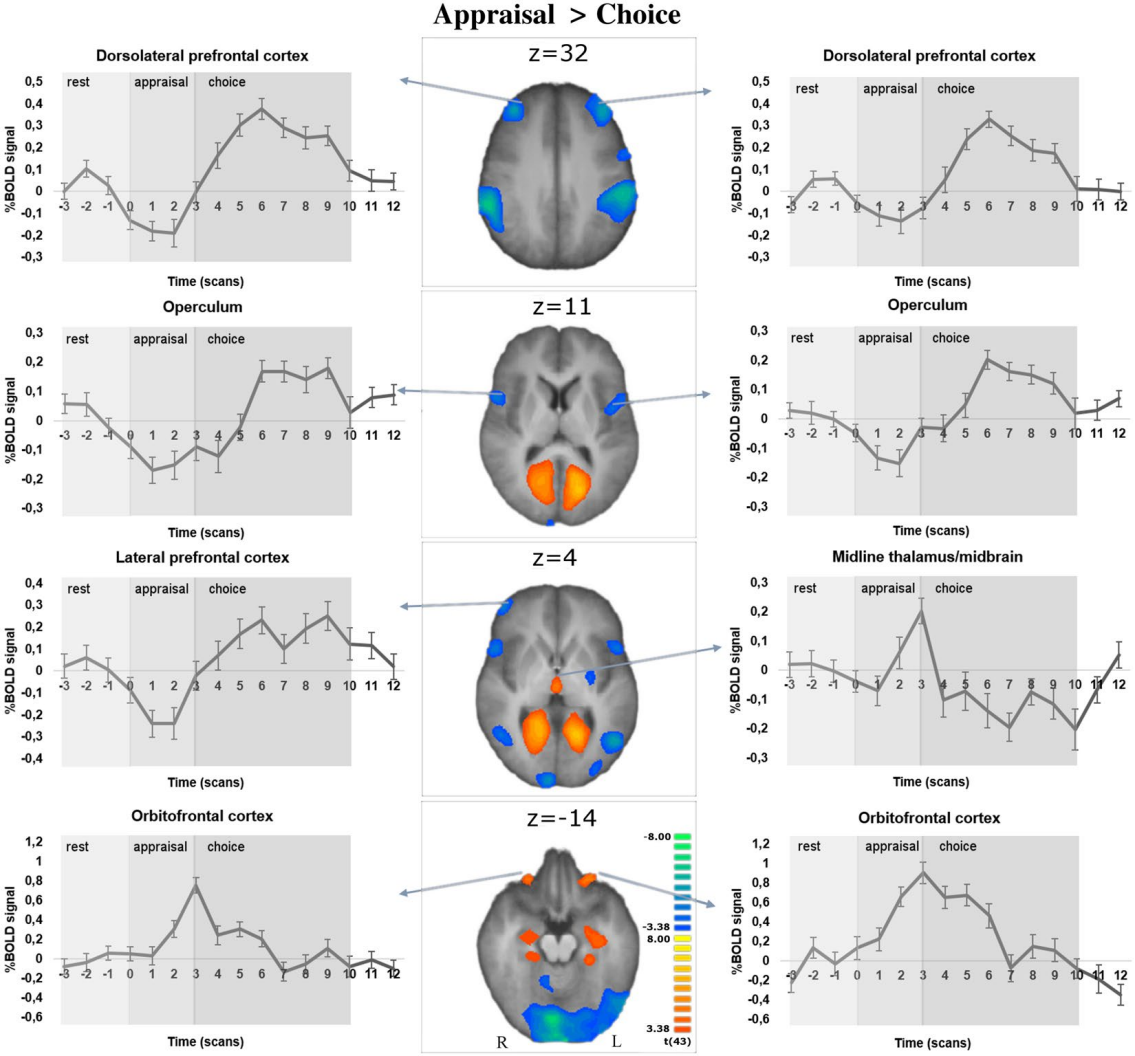
Contrast ‘appraisal’ vs. ‘choice’ (RFX, 44 participants, t(43) = 3.38, p < 0.01 FDR corrected). Time courses of BOLD signal changes are plotted for selected regions of interest.

To understand the neural basis of the distinct neurocognitive aspects of the task, we first contrasted the blocks of ‘appraisal’ (evaluation) with the blocks of ‘choice’ (appraisal > choice, RFX, t(43) = 3.38, p < 0.01 FDR corrected, Fig. 2 and Supplementary Table S1). Larger activation was found during ‘appraisal’ in the orbitofrontal cortex (OFC), entorhinal, posterior cingulate, midline thalamus/midbrain and left middle temporal gyrus. During ‘choice’ significant recruitment was observed in the anterolateral prefrontal cortex (PFC), right lateral PFC, frontal operculum, inferior parietal lobule and left putamen.

As revealed by the behavioural results, the participant often did not answer the most obvious option under the couple’s dyadic perspective of outcome maximization. To further investigate this issue, we performed an RFX analysis contrasting high cooperative vs low cooperative options.

**Figure 3:**
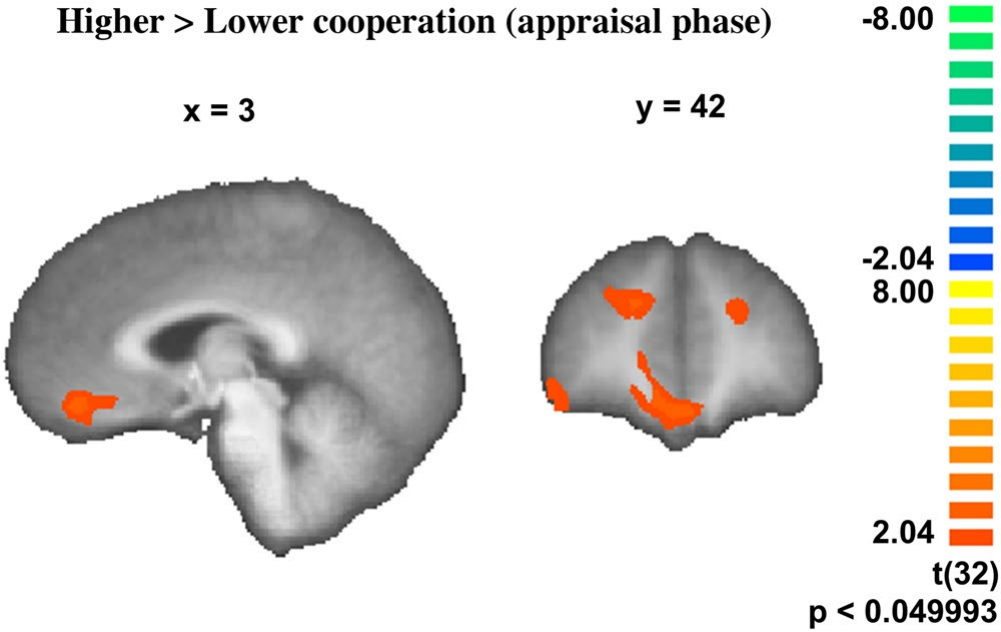
Contrast higher vs. lower cooperation during the ‘appraisal’ phase (RFX, n = 33, t(32) = 2.04, p < 0.05, minimum cluster extent 60).

We compared ‘appraisal’ periods which were concordant with the later choice of highest outcome for the couple with ‘appraisal’ blocks where the participant would later decide for an option of lower payoff for the couple (higher vs. lower cooperation, during appraisal). We found significant engagement of anterolateral PFC, OFC/ventromedial PFC, right ventrolateral PFC and the right temporal pole (Fig. 3 and Supplementary Table S2).

Concerning the contrast between higher and lower cooperation within the ‘choice’ blocks **(**higher vs. lower cooperation, during choice), we identified positive modulation in right precentral gyrus. Negative differences were found in subgenual cingulate cortex, left posterior orbitofrontal and entorhinal cortex (see Supplementary Fig. S2 and Supplementary Table S3).

**Figure 4:**
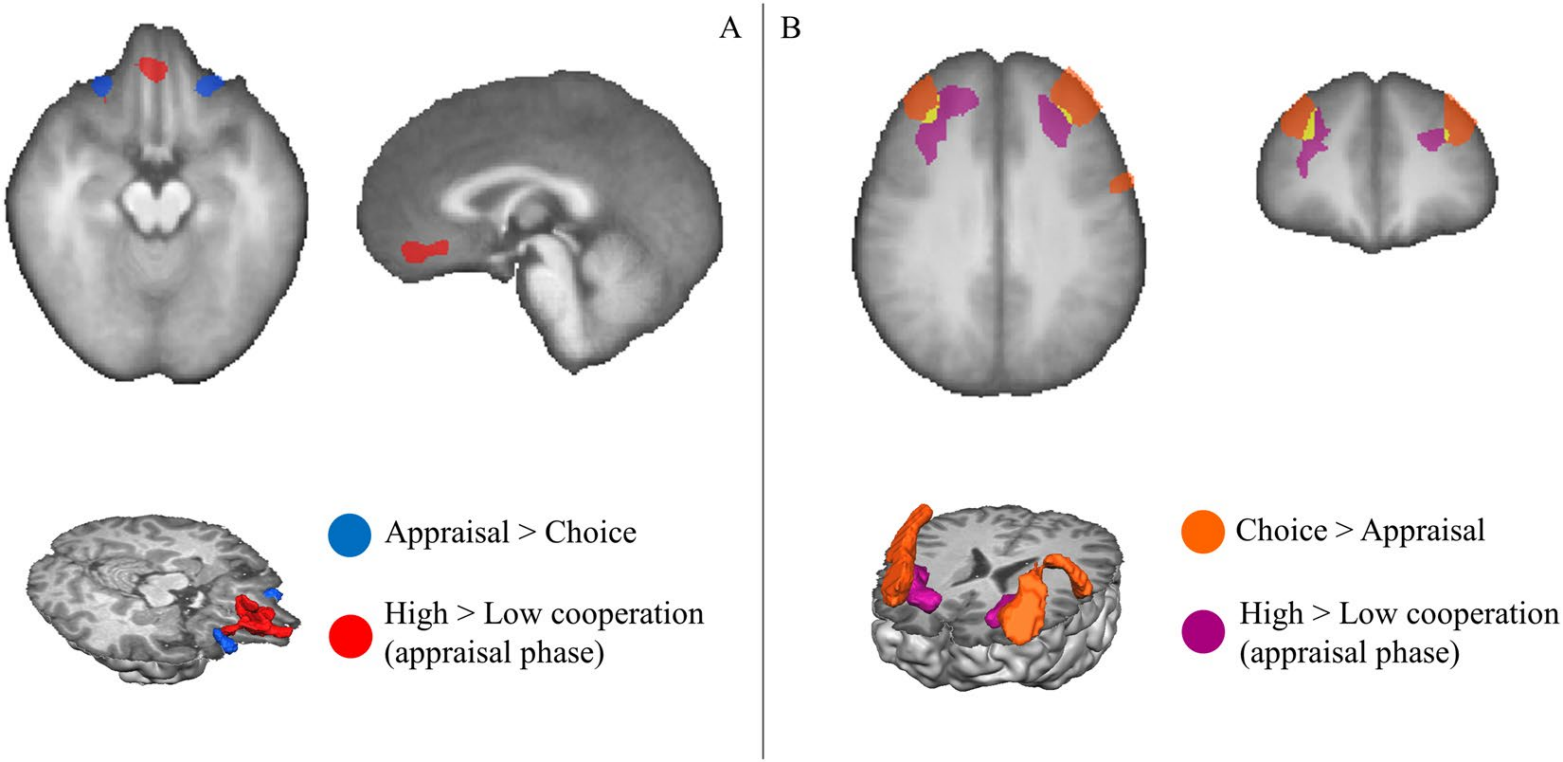
Summary: Clusters in the orbitofrontal and ventromedial PFC (**A**) and clusters in the anterolateral prefrontal cortex (**B**) identified at group-level in RFX analysis of various contrasts (see colour labels for each specific type of contrast in the insets).

Overall, our results suggest a novel functional parcellation of distinct regions in the orbitofrontal and in the prefrontal cortices. We summarize the involvement of the different subregions of the orbitofrontal cortex in Fig. 4A and prefrontal cortices in Fig. 4B, as reveal by the different contrasts described above. It is worth noting the appraisal (evaluation) vs. choice functional parcellation in the anterolateral prefrontal cortex.

## Discussion

This study sought to identify the neural correlates of a battle of the sexes dilemma, involving affective and hedonic conflict between romantic and “tribal” love. Here, the payoff matrices were explicitly calculated for each participant, part of a cohort in which football fandom features could be scored. These scores were found before to be correlated with activity in limbic and reward related structures^8^. We found a surprising pattern of behavioural responses in the affective and hedonic dilemmas studied here. The behavioural data showed that the participants often did not answer the most obvious option under the couple’s perspective of outcome maximization. This extends to the affective neuroscience of pair-bonding the finding, under non-affective conditions, that decision makers do not always act in accordance with standard predictions^29,30^ of rational utility maximization^29,31^ in terms of material or financial utility^15,32–34^. This seems to be indeed also the case for strong affective and hedonic dilemmas as studied here, confirming the notion of an extreme dilemma (‘tribal’ love vs. romantic love).

The cooperative behaviour in terms of the couple perspective is based on the inhibition of alternative prepotent conflicting actions (related to in-group bonding). Such a cooperative partner behaviour is mediated by motivated and goal-directed mechanisms for pair-bonding. Our work addresses for the first time the neural correlates of the strong affective dilemmas underlying the preference for cooperative behaviour in a long-lasting couple relationship. The main findings suggested a functional division of distinct frontal regions as a function of distinct emotional and cognitive aspects that emerge during the dilemma resolution. The lateral OFC was recruited in the appraisal of the emotional content of the dilemma. In this phase, the vmPFC likely encoded the reward value of the available options causing the dilemma, being related later on to the preference for cooperation. Surprisingly, the mesial part of the anterolateral PFC also activated in this initial phase, preferentially in the anticipation of cooperative actions. Later on, during deliberation, the lateral portion of the anterolateral PFC was recruited.

### A network orchestrating pair-bonding

The literature suggests that dorsolateral PFC, dorsal ACC and lateral OFC, which form cognitive and emotional control hubs, play a modulatory role over subcortical emotional/reward nodes during the deliberation of social dilemmas, in parallel with the social cognition network^26–28^. Here, we also found the involvement of anterior dlPFC and lateral OFC as expected, and further identified a temporal segregation of activity patterns, suggesting distinct roles during affective decision-making. In particular, we found the involvement of two distinct clusters in the anterolateral PFC for different contrasts (Fig. 4). A more mesial cluster activates for the contrast higher cooperation vs lower cooperation, early on in the ‘appraisal’ condition. A more lateral cluster in this region activates later on in the ‘choice’ condition and its role is independent of the affective components modulating the decision. The recruitment of this lateral cluster is related to the deliberative period, whereas lateral OFC activates as the participant faces and evaluates the dilemma.

The OFC is also thought to be involved in the computation of the expected affective value, along with the medial PFC and nucleus accumbens^22,35^. The idea that ventromedial PFC bears ‘storage’ of how much is a choice worth^36^ is in line with our results. In the present paradigm, vmPFC/medial OFC activates for the contrast high cooperation vs lower cooperation in the ‘appraisal’ condition (as the participant faces the dilemma), similarly to what is found in the right lateral PFC and the (more mesial part) anterolateral PFC. The results corroborate the idea of vmPFC as the core hub encoding reward values, in particular, as shown here, when affective content has a large weight. The vmPFC recruitment may be critical for the mechanisms underlying pair-bonding, as affective reward guides decision making. Also, **t**he anterior lateral PFC is thought to play a role in the inhibition of a prepotent choice^15,37^, which here may represent the selfish and less cooperative (from the point of view of the couple) option. Its recruitment directed by the willing to cooperate also suggests a role on the mechanisms that maintain pair-bonding and attachment.

### From emotional appraisal to rational choice

Limbic regions are strongly recruited early on as the participant faces the ‘battle of the sexes’ dilemma in the ‘appraisal’ period. Later, in the ‘choice’ condition, deliberation related regions show significant increases, suggesting a temporal dichotomy between emotional appraisal in the pre-decision phase and a more deliberative pattern during the ‘choice’ phase. Specifically, the results suggest that OFC and midline thalamus/midbrain are involved in the appraisal of the emotional content in the dilemma; while anterolateral PFC, right lateral PFC and frontal operculum dominate during the process of reasoning and risk/benefit weighting towards final decision-making.

### Cooperation in the battle of the sexes

In the present study, the dilemma is tailored to the participant’s preferences. The long-term nature of couple relationships guarantees that cooperative/non-cooperative patterns of behaviour are already conditioned by the mutual knowledge between the partners and their personal history, unlike most iterated game paradigms, where the history of the repeated interaction is confined to the day of scanning. In this way, we suggest that, as the participant faces the dilemma, there is an assessment of its emotional and reward content and an access to the stored affective and hedonic information. We found the recruitment of a cluster overlapping the midline thalamus and midbrain during the ‘appraisal’ condition. Dopamine containing areas in this region are also thought to support the computation of the expected reward value^35^. The identified activation pattern supports the idea that reward drive is prioritized and guides human decision-making^16^. In this way, we suggest that polar and anterolateral frontal regions (BA 9 and BA 10) receive the input from these emotion, hedonic processing and memory related areas, integrating this information in the process of reasoning and hedonic benefit weighting required in the ‘battle of the sexes’ dilemma.

Concerning limitations, the choice of sampling only male participants was to ensure homogeneity and because of the nature of the population (football fans). Future work should overcome this limitation by replicating our results in a female cohort.

In sum, in the present work, by identifying a distinct role for different subregions in prefrontal cortex, we shed new light on the neural mechanims that drive the willing to cooperate in ‘battle of the sexes’ dilemmas, when a in-grouping bonding conflict is present. This may contribute to a better understanding of the mechanisms underlying pair-bonding and the processing that support the orchestration and maintenance of adaptative social interactions.

## Methods

### Participants

The 44 participants were neuropsychologically characterized football fans of a team competing in the Portuguese First League. They were all males, aged between 21.3 and 60.2 years, 36.0 ± 11.2 (mean ± standard deviation, SD) and were involved in a romantic relationship of at least 1 year (mean 9.1 ± 8.4 years). Forty-three out of 44 participants used the joystick in the right hand. All the subjects had normal or corrected to normal vision.

Football fanaticism and team identification were first assessed using 2 self-report Likert scales (used in a 1 to 5 range)^38–40^. These scales were the Football Supporter Fanaticism Scale (FSFS, mean score and SD 3.09 ± 0.96, n = 44) and the Sport Spectator Identification Scale (SSIS, 4.07 ± 0.79, n = 44), which showed to be highly correlated (r(42) = 0.79).

Personality dimensions were assessed using a Portuguese Version^41,42^ of the Big Five Inventory (IGFP-5)^43^. This is a self-report inventory composed of 44 items (5-item Likert scale) to assess: openness to experience, conscientiousness, extraversion, agreeableness and neuroticism. The pool of participants included in the functional analysis scored 3.63 ± 0.46 (mean ± SD) in openness to experience; 3.62 ± 0.55 in conscientiousness; 3.44 ± 0.62 in extraversion; 3.69 ± 0.53 in agreeableness, and 2.62 ± 0.74 in neuroticism.

All subjects signed the informed consent for the present study, and experimental protocols were carried out in accordance with the Declaration of Helsinki and applicable regulations and guidelines. The study was approved by the Ethics Committee of the Faculty of Medicine of the University of Coimbra.

### Task

To determine participant specific payoff matrixes for the battle of the sexes dilemma, and before the MRI session, the participant (a football fan with formally evaluated fanaticism scores) answered two separate detailed questionnaires on his own or partner preferences concerning preferred activities. The forms contained a list of 38 activities/hobbies described in sentences like “Favourite band concert with the partner”, “Favourite band concert alone” or “Porto – Benfica in the final cup match in the stadium, with the partner”. The participant had to classify each statement in a 1 to 7 scale according to his own preferences in the first questionnaire. In the second questionnaire, he had to classify according to what he judged that would be his partner’s choice to each item. Each activity/hobby was always presented in two complementary forms: “alone” or “with the partner”, the latter meaning that in a given activity they would participate jointly as a couple.

The responses to these detailed questionnaires allowed for the construction of payoff matrixes, with explicit definition of outcome values. For instance, one could score the option “Have a dinner in your favourite restaurant with your partner” higher that the option “Have a dinner in your favourite restaurant alone”. Hence, the same activity/hobby could have a different outcome value in subsequent choice situations in which the participant engaged in either a lonely or a joint activity with the partner. In the example described in Fig. 1, one can see that the activity proposed by the participant (situation H1) provides a personal higher outcome value if his partner cooperates, by joining him (situation S0) than if she defects (situation S1). Hence, the answers to the questionnaires were used to create tailored outcome matrixes describing each particular dilemma.

These questionnaire responses and payoff matrixes were then used to assign difficult choice situations in our fMRI experimental task. Six tailored dilemmas were presented to each participant. The experiment had a boxcar design as described in Supplementary Fig. S1. In the first block of 9 seconds, the given dilemma was presented, corresponding to the ‘appraisal’ or evaluation period. Here we presented a participant’s preferred activity (always involving an important football match, given that participants were chosen as football fans) and his partner’s preferred and competing activity. This was followed by the ‘choice’ block where the participant faced the options and selected the decision. The individual block duration was further defined up to the participant’s response. Each pair of ‘appraisal’/‘choice’ periods was followed by a baseline period of 15 seconds. As described in Supplementary Fig. S1, when the subject faced the dilemma, he had to decide, between 4 options, the one that represents the most probable choice scenario: O1) each one does the favourite activity alone, O2) she gives up her preferred activity and goes with him, O3) he gives up his preferred activity and goes with her, and O4) they give up their preferred activity and do something else (with explicitly described activity) (Fig. 1).

### Acquisition parameters

Magnetic resonance imaging experiments were performed in a 3T Magnetom Trio Tim MRI scanner (Siemens, Erlangen, Germany) with a 12-channel head coil. A T1-weighted MPRAGE was measured with repetition time (TR) of 2530 ms, echo time (TE) of 3.42 ms, resolution 1 mm3, flip angle of 7°, matrix size 256 × 256, field of view of 256 × 256 and a slice thickness of 1 mm. To map and posteriorly correct the EPI sequence distortions, we acquired gradient field maps (GRE) before each EPI-BOLD sequence. Phase and magnitude field maps were acquired with the same orientation and the same field of view, TR of 3000 ms, TE of 30 ms, echo spacing 0.5 ms, phase resolution 100%, phase encoding direction from anterior to posterior, echo time difference of 2.46 ms and bandwidth in the phase direction of 31.25 Hz. Functional scanning was done using Echo Planar Imaging (EPI) sequences acquired a 100 times, with slice thickness of 3 mm and voxel size 4 mm2, 36 slices acquired parallel to the AC-PC line, TR 3000 ms, TE 30 ms, flip angle of 90°, matrix size 256 × 256 and FOV of 256 × 256.

Stimuli were displayed in an LCD monitor (NordicNeuroLab, Bergen, Norway) mounted ~156 cm away from the participants’ head and could be seen through a mirror mounted above the coil. The monitor has a frequency rate of 60 Hz and vertical vs horizontal dimensions of 698.40 × 392.85 mm. The subject could actively select the response using an MR-compatible joystick (Hybridmojo, San Mateo CA, USA).

### fMRI data analysis

Pre-processing of functional data was performed using BrainVoyager QX 2.8.2 (Brain Innovation, Maastricht, The Netherlands) as described elsewhere^8^. Data were corrected for geometrical distortion, slice scanning time differences, for motion, and filtered in the time domain. Anatomical and functional data were subsequently co-registered (and manually verified) and normalized according to the Talairach atlas. After the spatial normalization, spatial smoothing was performed using a Gaussian kernel of 8 mm FWHW. Statistical analysis was performed at group level using a General Linear Model (GLM) approach. The predictor’s model was obtained by convolution of the boxcar time course (the individual block duration was defined up to the participant’s response) with a two-gamma hemodynamic response function. Random effects analysis (RFX) was performed including all subjects (44 subjects) in the first analysis. These statistical maps were corrected for multiple comparisons using false discovery rate (FDR) with a fixed p-value of 0.01 (at the single voxel level) and minimum cluster extension of 18 contiguous voxels. Plots of percentage of BOLD change are shown triggered to the beginning of the ‘appraisal’ block. Time courses describe the trajectory of the hemodynamic response of the ‘appraisal’ condition, followed by the ‘choice’ condition, calculated in relation to the three last TRs of the ‘rest’ condition. In the subsequent analysis, the option for a model of random effects led to the exclusion of participants having one of the predictors equal to zero (i.e. the exclusion of participants who always pointed out the most cooperative option or who never pointed out this option). For these maps, a grey matter mask was obtained from the averaged anatomical file from all participants. Further correction for multiple comparisons was done using a cluster extent criterion, with a p value set at 0.05 and voxel extent estimation based on Monte Carlo simulations (1000 iterations). Only clusters larger than 60 contiguous voxels were considered.

## Electronic supplementary material

Supplementary Material

## Data Availability

Supporting material is available as supplementary images and tables. The dataset generated and analysed during the current study are available from the corresponding author on reasonable request.
