# Supplementary material for: The role of Prefrontal Cortex in a Battle of the Sexes Dilemma involving a Conflict between Tribal and Romantic love
Source: Sci Rep. 2018 Aug 14;8:12133. doi: 10.1038/s41598-018-30611-6 (PMC6092421; doi:10.1038/s41598-018-30611-6)
Supplement: Supplementary file 1 — Supplementary Material [file 41598_2018_30611_MOESM1_ESM.docx]

**Supplementary Material**

**The role of Prefrontal Cortex in a Battle of the Sexes Dilemma involving a Conflict between Tribal and Romantic love**

Isabel Catarina Duarte, Sónia Brito-Costa, Ricardo Cayolla, Miguel Castelo-Branco

**Figures:**


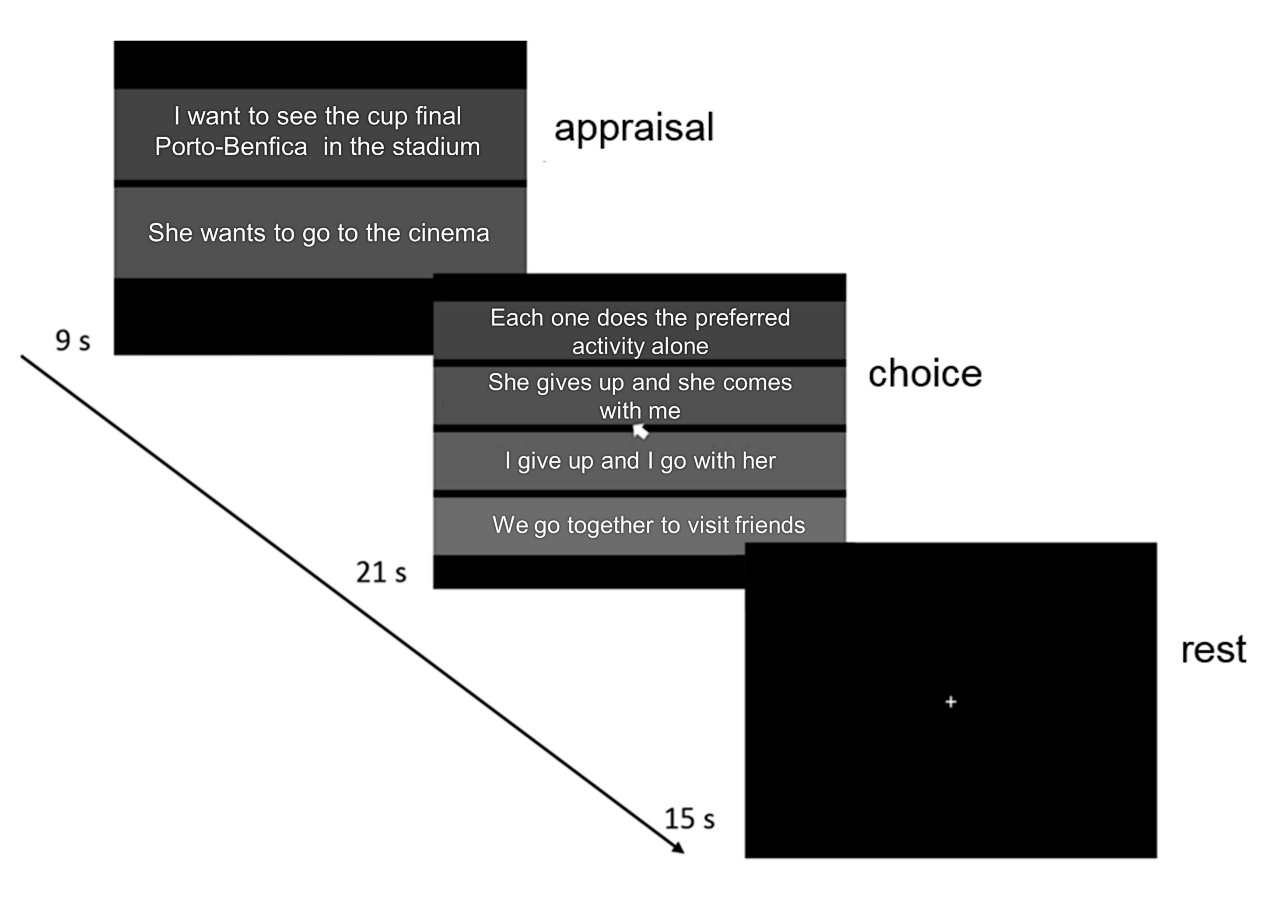


**Figure S1.** Experimental design. In the first block, the ‘appraisal’ condition, the participant reads the two competing components of the dilemma: 1) what he prefers to do (always involves the favorite team in a cup final match against a high rival team and it can be watched at home, in a café or in the stadium), and 2) what his partner prefers to do. In the following block, the participant has to choose the most probable scenario, according to the posed dilemma: O1) each one does the preferred activity alone, O2) she gives up her preferred activity and goes with him, O3) he gives up his preferred activity and goes with her and O4) they give up their preferred activity and do something else specified in that option. Each pair of ‘appraisal’/‘choice’ is followed by a fixation period.


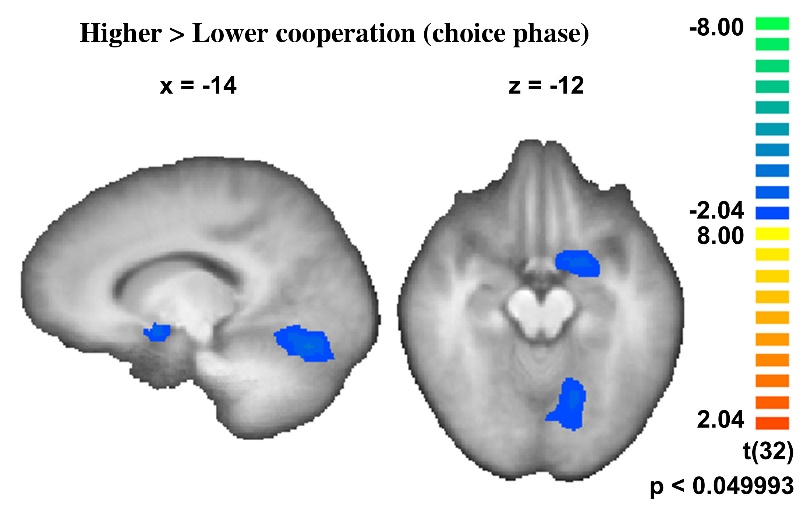


**Figure S2.** Contrast higher vs. lower cooperation during ‘choice’ phase. (RFX, n=33, t(32)=2.04, p<0.05, minimum cluster extent 60).

**Tables:**

**Table S1.** Regions revealed by the contrast of ‘appraisal’ vs. ‘choice’ conditions. Regions were identified from a whole-brain RFX-GLM analysis (t(43)=3.38, p<0.01 FDR corrected). The coordinates in Talairach space and the t and p values in the peak voxel describe the clusters.

|  |  | **Tal coordinates** | | |  |  |  |
| --- | --- | --- | --- | --- | --- | --- | --- |
|  | **H** | **x** | **y** | **z** | **t** | **p** | **#** |
| anterolateral prefrontal cortex (BA 9) | R | 36 | 41 | 34 | -5,85 | 0,000001 | 8814 |
| anterolateral prefrontal cortex (BA 9) | L | -39 | 41 | 34 | -5,76 | 0,000001 | 8802 |
| lateral prefrontal cortex (BA 10) | R | 45 | 47 | 4 | -4,65 | 0,000031 | 714 |
| orbitofrontal cortex (BA 11) | R | 27 | 35 | -14 | 4,59 | 0,000039 | 494 |
| orbitofrontal cortex (BA 11) | L | -27 | 35 | -17 | 5,30 | 0,000004 | 602 |
| operculum (BA 44) | R | 51 | 8 | 13 | -4,91 | 0,000014 | 3505 |
| operculum (BA 44) | L | -48 | 17 | 1 | -5,32 | 0,000004 | 3191 |
| entorhinal | R | 39 | -13 | -26 | 5,95 | <0,000001 | 7123 |
| entorhinal | L | -36 | -7 | -23 | 5,35 | 0,000003 | 4059 |
| putamen | L | -27 | -10 | 7 | -4,16 | 0,000149 | 494 |
| posterior cingulate (BA 30, 23, 31) | R,L | -15 | -70 | 13 | 6,98 | <0,000001 | 42117 |
| inferior parietal lobule | R | 57 | -37 | 31 | -7,14 | <0,000001 | 12368 |
| motor & parietal & inferior parietal lobule | R,L | -39 | -37 | 49 | -7,70 | <0,000001 | 52920 |
| middle temporal gyrus | L | -54 | -10 | -5 | 5,25 | 0,000005 | 854 |
| visual cortex and MT | R,L | 3 | -79 | -11 | -7,59 | <0,000001 | 65568 |
| MT | R | 51 | -52 | -2 | -5,86 | 0,000001 | 2902 |
| Midline thalamus/midbrain | R,L | 0 | -19 | 1 | 6,90 | <0,000001 | 1406 |

**Table S2.** Regions revealed by the contrast higher vs lower cooperation during appraisal (contrast between ‘appraisal’ conditions where the upcoming strategy was concordant with the option for highest outcome for the couple versus the option for a lower outcome) (RFX, t(32)=2.04, p<0.05, minimum cluster extent 60). The coordinates in Talairach space and the t and p values in the peak voxel describe the clusters.

|  |  | **Tal coordinates** | | |  |  |  |
| --- | --- | --- | --- | --- | --- | --- | --- |
|  | **H** | **x** | **y** | **z** | **t** | **p** | **#** |
| anterolateral prefrontal cortex (BA 9) | R | 33 | 23 | 31 | 3.06 | 0.004404 | 4441 |
| anterolateral prefrontal cortex (BA 9) | L | -24 | 32 | 28 | 2.70 | 0.010239 | 1735 |
| orbitofrontal/ventromedial prefrontal cortex (BA 11, 12) | R,L | 3 | 44 | -11 | 3.25 | 0.002718 | 4141 |
| ventrolateral prefrontal cortex (BA 11, 10) | R | 45 | 50 | -3 | 3.19 | 0.003207 | 2522 |
| temporal pole | R | 52 | 8 | -32 | 4.00 | 0.00035 | 13956 |
| angular gyrus | R | 36 | -79 | 31 | -2.66 | 0.012126 | 2417 |
| angular gyrus | L | -30 | -85 | 25 | -3.11 | 0.003932 | 3463 |
| middle temporal/occipital | L | -66 | -47 | 10 | -3.74 | 0.000726 | 2525 |

**Table S3** Regions revealed by the contrast higher cooperation vs lower cooperation (contrast between ‘choice’ conditions where the strategy was concordant with the option for highest outcome for the couple versus the option for a lower outcome) (RFX, t(32)=2.04, p<0.05, minimum cluster extent 60). The coordinates in Talairach space and the t and p values in the peak voxel describe the clusters.

|  |  | **Tal coordinates** | | |  |  |  |
| --- | --- | --- | --- | --- | --- | --- | --- |
|  | **H** | **x** | **y** | **z** | **t** | **p** | **#** |
| posterior orbitofrontal cortex (BA 25, 34) | L | -21 | 5 | -11 | -3.40 | 0.001814 | 1907 |
| temporal pole | R | 30 | 11 | -20 | -3.64 | 0.000962 | 1645 |
| inferior parietal lobule/occipital gyrus | L | -49 | -79 | 19 | -3.70 | 0.000804 | 8092 |
| lingual gyrus | L | -15 | -70 | -17 | -3.69 | 0.000838 | 8671 |
| precuneus | R,L | 0 | -67 | 59 | -3.23 | 0.002839 | 2260 |
| precentral gyrus | R | 12 | -25 | 67 | 2.67 | 0.011705 | 1846 |
